# POTENTIALLY PREVENTABLE HOSPITALIZATIONS IN A CONNECTICUT MEDICAID HOME AND COMMUNITY-BASED SERVICES POPULATION

**DOI:** 10.1093/geroni/igae098.3533

**Published:** 2024-12-31

**Authors:** Ellis Dillon, Richard Fortinsky, Lisa Barry, Chae Man Lee, Martha Porter, Deborah Migneault, Julie Robison

**Affiliations:** University of Connecticut, Farmington, Connecticut, United States; University of Connecticut, Farmington, Connecticut, United States; UConn Center on Aging, Farmington, Connecticut, United States; University of Connecticut, Farmington, Connecticut, United States; University of Connecticut, Farmington, Connecticut, United States; University of Connecticut Health, Farmington, Connecticut, United States; University of Connecticut, Farmington, Connecticut, United States

## Abstract

State Medicaid programs address the needs of older adults, and people with disabilities or chronic illnesses through a range of home and community-based services (HCBS) and aim to prevent adverse health outcomes. This study determines rates of potentially preventable hospitalizations (PPH) for Connecticut residents receiving Medicaid-funded HCBS and compares rates across HCBS programs using a CMS Home Health quality measure. This retrospective cohort analysis linked Medicaid and Medicare fee-for-service claims, Medicaid administrative data, and uniform clinical and functional assessment data for 33,688 Connecticut residents. PPH rates between 4/1/2022-9/30/2022 were compared across six HCBS programs via logistic regressions adjusted for the 8-point InterRAI Risk of Hospital Use Scale (Hosp-RiskHC) score. Rates of PPHs ranged from 0.8% (Autism Waiver) to 15.7% (Personal Care Attendant Waiver, PCA). Individuals with PPHs represented over half (55.6%) of all hospitalized individuals. PCA participants were more likely (OR=1.3, 95% CI:1.13-1.58) and Acquired Brain Injury Waiver participants were less likely (OR=0.7, 95% CI:0.48-0.93) to have a PPH compared to Elder Waiver recipients. Likelihood of experiencing a PPH increased with each successive Hosp-RiskHC score (OR=1.2, 95% CI:1.16-1.22). Controlling for risk factors, rates of PPH varied across HCBS programs, potentially due to the nature of their disabilities, differing services provided by each program, or a combination of individual and structural factors. Identifying variations in PPH rates is a critical step toward building a Value-Based Payment (VBP) program for HCBS providers, who have historically been excluded from VBP opportunities, and identifying systemic gaps and opportunities for coordinated interventions across healthcare providers.

